# Flattening Patterns of Antimicrobial Resistance Levels in Indicator *E. coli* in Dutch Livestock

**DOI:** 10.1111/zph.70025

**Published:** 2025-11-16

**Authors:** Anita Dame‐Korevaar, Erik Kuiper, Jose L. Gonzales, Kees Veldman

**Affiliations:** ^1^ Wageningen Bioveterinary Research Lelystad the Netherlands

**Keywords:** antimicrobial resistance, antimicrobial usage, livestock, trend analysis

## Abstract

**Introduction:**

Antimicrobial resistance (AMR) is defined by the World Health Organization as one of the most important health threats, that needs a One Health approach. Monitoring AMR in livestock is an important element, which has been done in the Netherlands in a monitoring program since 1998. The aim was to analyse AMR trends during the periods 2010–2018 and 2019–2023.

**Methods:**

A dataset containing the antimicrobial resistance data of > 12,000 indicator *E. coli* isolates collected from faecal samples from broilers, fattening pigs and veal calves at slaughter houses, as part of the Dutch AMR monitoring program, was built to analyze AMR trends. ECOFF values were used to distinguish wild‐type (WT) and non‐wild‐type (non‐WT, phenotypically resistant) isolates.

**Results:**

In the period 2010–2018 decreasing resistance patterns to most antibiotics were seen in broilers, fattening pigs and veal calves. However, in the period 2019–2023 flattening resistance patterns were observed in broilers and fattening pigs for antibiotics amoxicillin/ampicillin, sulfamethoxazole and trimethoprim, at relatively high levels of resistance, despite a reduction in antibiotic usage during this period.

**Conclusions:**

Following a significant decreasing trend in the prevalence of AMR between 2010 and 2018, no significant changes in the prevalence of AMR were observed between 2019 and 2023 for most antibiotics. To get more insight into the limited correlation between usage and resistance in recent years, further studies are needed to analyse the relation, and underlying factors, between antibiotic usage and AMR more in depth.

**Summary**

- The results show that following a decreasing trend in prevalence of antimicrobial resistance (AMR) in livestock in the period 2010–2018, AMR prevalence seem to flatten in the period 2019–2023, whereas antimicrobial usage (AMU) still decreased in the same period.
- This apparent lack of correlation between AMU and AMR raises questions and indicates the need for more insight in this relation between AMU and AMR and underlying factors.
- After the successful efforts made by farmers, veterinarians, policymakers and other stakeholders to reduce AMR, this study shows that we need to improve our understanding of the complex relation between AMU and AMR to identify possible additional measures to further reduce AMR in livestock.

## 1 Introduction

Antimicrobial resistance (AMR) is defined by the World Health Organization as one of the most important health threats (WHO 2023). The presence of antimicrobial resistant bacteria in humans, animals and the environment, driven by antimicrobial usage (AMU) in both animals and human populations (Allel et al. 2023; Bell et al. 2014; Holmes et al. 2016) and the transmission of AMR between these reservoirs, has resulted in a public and animal health challenge that needs a systematic One Health approach, taking into account the different sectors (Arnold et al. 2024). An important tool to address this One Health issue is the monitoring of AMR in livestock. Monitoring of AMR in food‐producing animals and food is needed to generate reliable data on the occurrence and spread of AMR, which can be used to inform policy makers and develop measures to control this.

According to Commission Implementing Decision (EU) 2020/1729, monitoring of AMR is mandatory in *Salmonella* spp., *Campylobacter coli* ( *C. coli* ), *Campylobacter jejuni* ( *C. jejuni* ) and indicator *Escherichia coli* ( *E. coli* ), in livestock and meat (European Food Safety Authority (EFSA); European Centre for Disease Prevention and Control (ECDC) 2023). In the Netherlands, monitoring of AMR started already in 1998 in broilers and results are yearly presented in the report Monitoring of Antimicrobial Resistance and antibiotic usage in Animals in the Netherlands (MARAN) (de Greeff et al. 2023). The high use of antimicrobials in livestock together with increasing levels of AMR, and the presence of livestock‐associated methicillin‐resistant *Staphylococcus aureus* (LA‐MRSA) and extended spectrum beta‐lactamase‐producing bacteria (ESBLs) in livestock and humans led to a public health debate. As a consequence, a combination of compulsory and voluntary measures was implemented in the Dutch livestock sector in 2009/2010 (Speksnijder et al. 2015). This resulted in a more than 76% reduction of sales of antimicrobials in 2023 compared to the reference year 2009 (SDa 2024a) which coincided with decreasing trends of AMR since the implementation of these policy changes (de Greeff et al. 2023; Hesp et al. 2019).

Hesp et al. (2019) analysed the AMR trends in indicator *E. coli* before and after the reference year of 2009, when the policy changes were implemented. Using Dutch monitoring data from 1998 to 2016, it was observed that AMR trends in broilers and pigs significantly increased before the reference year 2009 followed by a significant decrease in the years 2010–2016. In this analysis commensal *E. coli* showed to be a useful indicator to detect trends in AMR (Hesp et al. 2019). Following this study, efforts were made to automize the visualisation and statistical analysis of AMR monitoring data in the Netherlands, aiming to optimise the reporting and interpretation of monitoring results included in the yearly MARAN reports. During the last years, the initial decreasing trends in resistance seem to have flattened, despite decreasing antibiotic usage until 2022 (SDa 2023). Therefore, the aim of this study was to assess the trends in AMR since 2010 to 2023 and to reflect on the long‐term effect of the ongoing decreasing antibiotic usage on AMR in livestock.

## 2 Material and Methods

### 2.1 Animal Sampling and Bacterial Isolation

As part of the Dutch AMR monitoring program, faecal samples are collected from broilers, pigs, and veal calves at slaughter houses, as described by Hesp et al. (2019). For every animal species, about 300 *E. coli* isolates are annually isolated from 300 faecal samples by direct inoculation on MacConkey agar plates followed by randomly selecting one typical *E. coli* ‐suspected colony per sample (Hesp et al. 2019) which is purely cultured on blood agar plates. After identification, confirmed *E. coli* cultures are suspended in peptone‐glycerol medium and stored at −80°C pending analysis.

### 2.2 Susceptibility Testing

The bacterial isolates included in this dataset were tested at the Dutch National Reference Laboratory on Antimicrobial resistance (NRL‐AR, WBVR). Susceptibility testing was performed with broth microdilution according to ISO guidelines (ISO 20776‐1). For the interpretation of the data in this study the same epidemiological cut‐off (ECOFF) values were used for all years in the dataset, using the values as defined by the European Committee on Antimicrobial Susceptibility Testing (EUCAST) (EUCAST 2024) and EU Decision 2020/1729 (European Union 2020) and presented in Table 1, to distinguish between wild‐type (WT) and non‐wild type (non‐WT, phenotypically resistant) isolates. In this paper “non‐WT” versus “WT” refers to individual isolates, “resistance” refers to the situation of antimicrobial resistance at the population level, i.e., Dutch livestock, and the changes in trends of AMR. Minimum inhibitory concentration (MIC) data were interpreted and transformed to binary data using 1 (non‐WT, MIC > ECOFF value) and 0 (WT, MIC ≤ ECOFF) and consequently, the proportion and percentage of resistant isolates were determined for every individual antibiotic included in the currently used panel according to EFSA technical guidelines (EFSA Authority EFS et al. 2019).

**TABLE 1:** Epidemiological cut‐off (ECOFF) values used in the analysis to distinguish wild‐type (WT) and non‐wild‐type (non‐WT, phenotypically resistant) isolates. ECOFFs were based on EUCAST 2024 and EU Decision 2020/1729 2020. Minimum inhibitory concentration (MIC) data were interpreted and transformed to binary data using 1 (non‐WT (phenotypically resistant), MIC > ECOFF value) and 0 (WT, susceptible, MIC ≤ ECOFF).

Antibiotic	ECOFF‐value
Amoxicillin	8
Amikacin	8
Ampicillin	8
Azithromycin	16
Cefotaxime	0.25
Ceftazidime	1
Chloramphenicol	16
Ciprofloxacin	0.06
Colistin	2
Doxycycline	8
Gentamicin	2
Meropenem	0.06
Nalidixic acid	8
Sulfamethoxazole	64
Tetracycline	8
Tigecycline	0.5
Trimethoprim	2

### 2.3 Dataset

The dataset used for this study contained MIC values from indicator *E. coli* isolates obtained from broilers, fattening pigs and veal calves in Dutch slaughterhouses between 1998 and 2023. The antimicrobial panels have changed over the years; therefore we selected the antibiotics that are currently present in the antimicrobial panel: amikacin, ampicillin (formerly amoxicillin), azithromycin, chloramphenicol, ciprofloxacin, colistin, tetracycline (formerly doxycycline), cefotaxime, ceftazidime, gentamicin, meropenem, nalidixic acid, sulfamethoxazole, tigecycline, trimethoprim. In two cases, data of two antibiotics from the same antimicrobial class were combined, as they were consecutively present in the panel and are considered to show complete cross‐resistance: (a) amoxicillin and ampicillin; (b) doxycycline and tetracycline. Most of the antibiotics were present in the antimicrobial panel since 2010, except for amikacin, azithromycin, meropenem, tigecycline. For these antibiotics the available years were included in the dataset.

After harmonising the datasets including the years since the start of the monitoring program (1998–2023), a subset of the dataset (2010–2023) was made to assess resistance trends since the year of the policy change (2010–2018) and for the recent period, arbitrarily set at the recent 5 years (2019–2023). The final dataset (2010–2023) included 4342 isolates from broilers, 4237 isolates from pigs, 3931 isolates from veal calves.

### 2.4 Statistical Analysis

Statistical analysis was based on the methods described by Hesp et al. (2019). In short, the proportion of non‐WT isolates (*R*) for every livestock species (*i*) and antibiotic $\left(j\right)$ was modelled by fitting a generalised linear model with a Poisson error distribution:

$$R_{\mathrm{ij}}=\propto +\mathrm{\beta x}+\text{offset}\left(\ln\left(N_{\mathrm{ij}}\right)\right)+\varepsilon$$

where response variable $R_{\mathrm{ij}}$ is the number of non‐WT isolates, ∝ is the model intercept, x is the time in years, as numerical variable, $N_{\mathrm{ij}}$ is included as offset, representing the total number of tested isolates for livestock species (*i*) and antibiotic $\left(j\right)$. By introducing $N_{\mathrm{ij}}$ as offset the proportion of non‐WT isolates (*R*) is estimated. Finally, ε is the residual error. For one set of models the variable year consisted of the period between 2010 and 2018, for the second set of models the period between 2019 and 2023. β is the fitted parameter describing the rate of change in proportion of R in time, with $\exp\left(\beta \right)$ interpreted as the incidence rate ratio (IRR) of R for each combination of antibiotic and animal species. Analysis was performed in R software, version 4.3.3. (Dorado‐García et al. 2016).

## 3 Results

**FIGURE 1:**
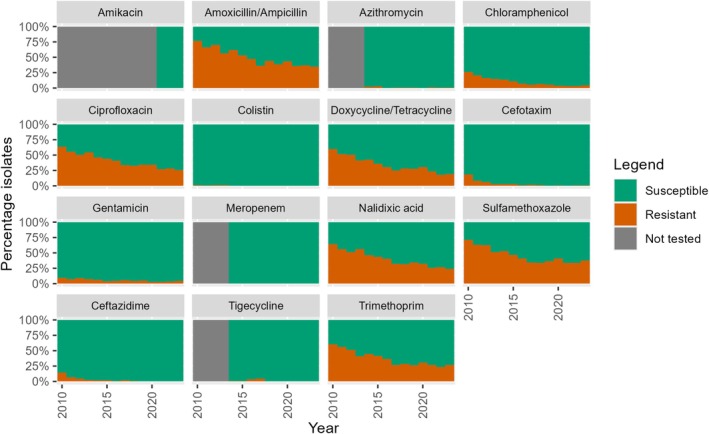
Percentage of non‐WT indicator *E. coli* isolates against different antibiotics, isolated from faecal samples from broilers in the period 2010–2023 in the Netherlands.

**FIGURE 2:**
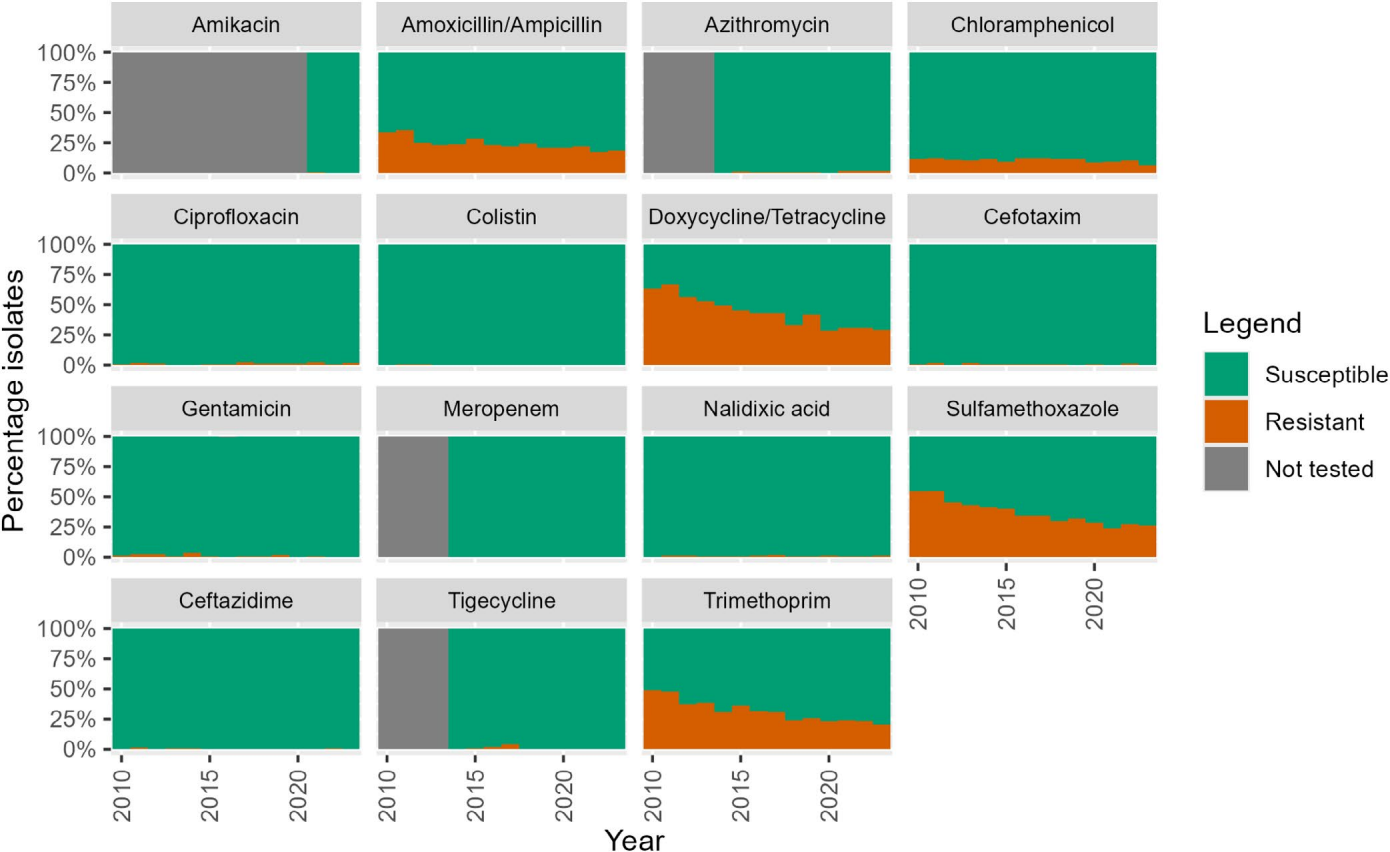
Percentage of non‐WT indicator *E. coli* isolates against different antibiotics, isolated from faecal samples from pigs in the period 2010–2023 in the Netherlands.

**FIGURE 3:**
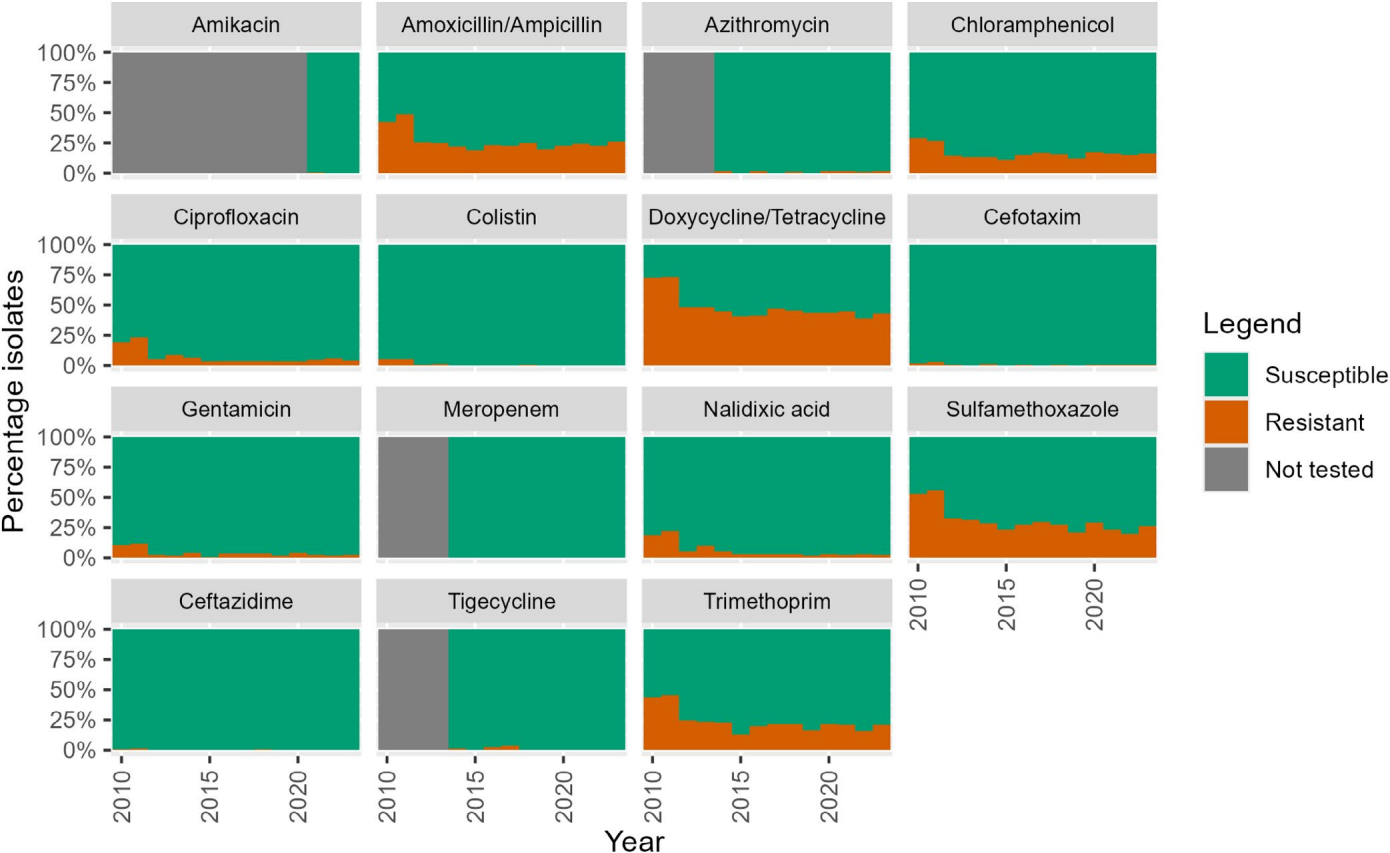
Percentage of non‐WT indicator *E. coli* isolates against different antibiotics, isolated from faecal samples from veal calves in the period 2010–2023 in the Netherlands.

The percentage of non‐WT indicator *E. coli* isolates from broilers, fattening pigs and veal calves collected between 2010 and 2023 against different antibiotics is presented in Figures 1, 2, 3 (de Greeff et al. 2024). Results of the analysis of the resistance percentage between 2010 and 2018 versus the resistance percentage in the recent 5 years (2019–2023) are presented in Table 2.

**TABLE 2:** Incidence rate ratio (IRR, 95% CI) and annual prevalence (%, min–max) of resistant *E. coli* isolates against different antibiotics in the period 2010–2018 and 2019–2023 from faecal samples from broilers, fattening pigs and veal calves in the Netherlands. Bold numbers indicate a significant increasing (IRR > 1) or decreasing (IRR < 1) trend in the percentage of resistant isolates. For the antibiotics with a missing IRR the trend analysis could not be performed due to a lack of data or insufficient number of resistant isolates.

	2010–2018					2019–2023				
	IRR			Annual prevalence (%)		IRR	95% CI (2.5–97.5)		Annual prevalence (%)	
		95% CI (2.5–97.5)		Min	Max				Min	Max
*Broilers*										
Amikacin				0.00	0.00				0.00	0.00
Amoxicillin/Ampicillin	**0.92**	0.90	0.94	36.21	76.41	0.96	0.91	1.02	34.60	43.61
Azithromycin				0.00	2.50	1.02	0.62	1.68	0.00	1.00
Chloramphenicol	**0.83**	0.80	0.87	5.65	26.06	0.95	0.79	1.14	3.00	5.13
Ciprofloxacin	**0.92**	0.90	0.94	32.55	63.73	**0.92**	0.86	0.99	25.61	34.43
Colistin	**0.61**	0.41	0.81	0.00	1.06				0.00	0.00
Doxycyline/Tetracycline	**0.90**	0.88	0.92	24.92	59.86	**0.88**	0.82	0.95	18.00	30.16
Cefotaxim	**0.68**	0.62	0.74	1.00	18.31	1.45	0.63	4.09	0.00	0.35
Gentamicin	**0.91**	0.86	0.96	4.00	9.51	0.98	0.82	1.18	2.67	5.13
Meropenem				0.00	0.00				0.00	0.00
Nalidixic acid	**0.92**	0.90	0.94	31.54	64.08	**0.91**	0.85	0.97	23.88	34.29
Sulfamethoxazole	**0.91**	0.89	0.93	33.56	71.13	0.99	0.93	1.05	33.67	40.98
Ceftazidime	**0.68**	0.62	0.74	0.67	14.44	2.97	0.85	44.83	0.00	0.35
Tigecycline				0.00	4.32				0.00	0.00
Trimethoprim	**0.91**	0.89	0.93	26.58	60.56	0.98	0.91	1.05	23.67	30.82
*Pigs*										
Amikacin				0.00	0.00				0.00	0.67
Amoxicillin/Ampicillin	**0.95**	0.93	0.98	22.00	35.54	0.95	0.88	1.03	17.06	22.33
Azithromycin				0.00	1.01	1.39	0.97	2.06	0.00	1.67
Chloramphenicol	1.00	0.96	1.05	9.40	12.71	0.90	0.80	1.01	6.33	11.84
Ciprofloxacin	1.06	0.86	1.32	0.00	2.00	1.06	0.76	1.48	0.33	2.00
Colistin	0.51	0.13	1.01	0.00	0.70				0.00	0.00
Doxycyline/Tetracycline	**0.93**	0.91	0.95	32.89	66.90	**0.93**	0.87	1.00	28.81	41.78
Cefotaxim	0.84	0.68	1.03	0.00	1.74	1.43	0.44	5.93	0.00	1.00
Gentamicin	0.85	0.68	1.03	0.00	3.57	**0.37**	0.15	0.71	0.00	1.97
Meropenem				0.00	0.00				0.00	0.00
Nalidixic acid	1.03	0.89	1.21	0.35	1.67	1.13	0.77	1.68	0.66	1.33
Sulfamethoxazole	**0.93**	0.91	0.95	29.90	54.70	0.95	0.89	1.02	24.00	31.91
Ceftazidime	0.86	0.66	1.09	0.00	1.05	1.20	0.50	3.22	0.00	0.67
Tigecycline				0.00	4.00				0.00	0.00
Trimethoprim	**0.93**	0.90	0.95	24.25	48.94	0.96	0.89	1.03	20.33	25.99
*Veal calves*										
Amikacin				0.00	0.00				0.00	0.65
Amoxicillin/Ampicillin	**0.92**	0.88	0.97	18.77	48.80	1.05	0.97	1.13	20.07	25.75
Azithromycin				0.00	1.71	1.33	0.95	1.92	0.00	1.67
Chloramphenicol	**0.94**	0.88	0.99	11.60	29.07	1.03	0.94	1.13	12.59	17.33
Ciprofloxacin	**0.78**	0.71	0.85	3.41	23.49	1.12	0.94	1.33	3.33	6.25
Colistin	**0.49**	0.36	0.63	0.00	5.42				0.00	0.00
Doxycyline/Tetracycline	**0.94**	0.91	0.97	40.61	73.49	0.99	0.94	1.05	39.14	44.44
Cefotaxim	**0.74**	0.59	0.91	0.00	3.01	1.22	0.65	2.47	0.00	0.65
Gentamicin	**0.86**	0.75	0.99	0.68	11.45	0.95	0.75	1.20	1.64	4.00
Meropenem				0.00	0.00				0.00	0.00
Nalidixic acid	**0.75**	0.68	0.82	2.65	22.29	1.07	0.85	1.35	1.70	2.96
Sulfamethoxazole	**0.92**	0.88	0.96	23.55	56.02	1.01	0.93	1.10	19.74	28.67
Ceftazidime	0.93	0.65	1.32	0.00	1.20	1.76	0.74	6.11	0.00	0.33
Tigecycline				0.00	3.32				0.00	0.33
Trimethoprim	**0.90**	0.85	0.96	12.97	45.18	1.01	0.93	1.10	16.12	21.33

### 3.1 Broilers

The results show that the percentage of non‐WT isolates from broilers significantly decreased between 2010 and 2018 for all antibiotics. Trends could not be estimated for azithromycin, amikacin, meropenem and tigecycline, due to a lack of data or insufficient numbers of non‐WT isolates. However, for the recent 5 years, the period 2019–2023, most antibiotics did not show a significant change in the percentage of non‐WT isolates, except for ciprofloxacin, doxycycline/tetracycline and nalidixic acid; they showed a significant decrease in prevalence. Regarding the antibiotics with flattening patterns, chloramphenicol and gentamicin both reached low prevalence levels (with maximum levels around 5%); however, antibiotics amoxicillin/ampicillin, sulfamethoxazole and trimethoprim show flattening patterns although levels of resistance are still relatively high with levels above 20% (trimethoprim) – 30% (amoxicillin/ampicillin and sulfamethoxazole). No trends for the last 5 years could be estimated for colistin. There was a low prevalence of non‐WT isolates to colistin, but also to azithromycin, cefotaxime and ceftazidime (Table 2, Figure 1).

### 3.2 Pigs

Also for fattening pigs significant decreasing trends in resistance are observed in the period 2010–2018 for antibiotics amoxicillin/ampicillin, doxycycline/tetracycline, sulfamethoxazole and trimethoprim. Trends could not be estimated for amikacin, azithromycin, meropenem and tigecycline, due to a lack of data or insufficient number of non‐WT isolates. In the period 2019–2023 most of the initially decreasing patterns of these antibiotics flattened: amoxicillin/ampicillin, sulfamethoxazole and trimethoprim did not show any significant decrease in resistance, although the prevalence of non‐WT isolates is still relatively high, between 20% and 30%. Antibiotics doxycycline/tetracycline and gentamicin do show decreasing patterns in the period 2019–2023; however for gentamicin the prevalence is low (< 2%) and for doxycycline/tetracycline the resistance seems to level off after 2020. No trends for the last 5 years could be estimated for colistin, having a low prevalence of non‐WT isolates (Table 2, Figure 2).

### 3.3 Veal Calves

Veal calves show a significant decreasing percentage of resistance in the period 2010–2018 for most antibiotics except for ceftazidime. Trends could not be estimated for amikacin, azithromycin, meropenem and tigecycline, due to a lack of data or an insufficient number of non‐WT isolates. In the period between 2019 and 2023, no significant change in the prevalence of non‐WT isolates was observed. Although some of the antibiotics reached a low prevalence (ciprofloxacin, cefotaxime, gentamicin, nalidixic acid), the prevalence of non‐WT isolates is still moderate to high for chloramphenicol (between 13% and 17%), doxycycline/tetracycline (39%–44%), sulfamethoxazole (20%–29%) and trimethoprim (16%–21%). In the period 2019–2023 amoxicillin/ampicillin and ciprofloxacin even tend to increase, although this is not significant. No trends for the last 5 years could be estimated for colistin, having a low prevalence of non‐WT isolates (Table 2, Figure 3).

## 4 Discussion

The results of the analysis show that after a significant decrease in the percentage of resistance to most of the antibiotics in the period 2010–2018, in the recent 5 years (2019–2023) flattening of the AMR patterns is observed. Although for some of the antibiotics the flattening trend can be explained by reaching low, close to 0%, levels of resistance, for other antibiotics the leveling‐off occurs at relatively high prevalence of resistance. Resistance levels against antibiotics amoxicillin/ampicillin, sulfamethoxazole and trimethoprim are stabilising in both broilers and pigs, with resistance levels between 27% and 37% in broilers and between 18% and 26% in pigs in the year 2023. The observation of flattening AMR patterns raises questions, especially in the context of AMU in the same livestock sectors, based on the expected relation between AMU and AMR (Allel et al. 2023; Bell et al. 2014; Holmes et al. 2016). Antibiotic usage in the Netherlands decreased until 2022 in both broilers and pigs, as reported by the Netherlands Veterinary Medicines Institute (SDa) (SDa 2023). SDa yearly reports AMU in different livestock sectors, based on the defined daily dosage per animal (DDDA_NAT_), which expresses the amount of antibiotics used within a specific livestock sector in the Netherlands (SDa 2023). There is some variation in usage between the different years and the reduction of antibiotic usage might not follow a linear slope; however in general the use of antibiotics continued to decrease in the period 2019–2022, although data from 2023 was comparable to 2022 (SDa 2024a). Contrary to the stabilising AMR patterns, in the period 2019–2023 aminopenicillin usage in broilers reduced from 5.37 to 3.39 DDDA_NAT_ (SDa 2024b). The use of trimethoprim/sulfonamides in broilers reduced from 0.78 to 0.39 and in pigs from 1.01 to 0.82 DDDA_NAT_ (SDa 2024b). The same pattern is seen for doxycycline/tetracyclines in pigs, of which resistance levels tend to stabilise after 2019, whilst antibiotic usage decreased from 3.54 to 2.05 DDDA_NAT_ (SDa 2024b).

Also in veal calves, the percentage of isolates resistant to amoxicillin/ampicillin, sulfamethoxazole and trimethoprim is flattening in the recent 5 years. In addition to these antibiotics also chloramphenicol and doxycycline/tetracycline show flattening patterns at relatively high levels of resistance. Contradictory to the broiler and pig sector, antibiotic usage in the veal sector did not decrease continuously during the recent years, with a DDDA_NAT_ varying between 15.23 and 16.44 in the period 2019–2023 (SDa 2024b). The apparent lack of correlation during the recent years between antibiotic usage and resistance is raising questions. Flattening patterns in a situation where resistance levels are stable over time and at a low level, as is observed in broilers for, for example, chloramphenicol and gentamicin, make sense and major changes in these favourable situations, as is seen in some other countries, are not expected (European Food Safety Authority (EFSA); European Centre for Disease Prevention and Control (ECDC) 2024). However, it is striking, especially for amoxicillin/ampicillin and trimethoprim/sulfonamides, with relatively high levels of AMR, that further reduction in antibiotic usage does not lead to a further reduction in resistance levels. Even if the magnitude of the decrease of antibiotic usage is reducing for these antibiotic classes, as is indicated by the figures from 2023 (SDa 2024a), it is remarkable that flattening of the antibiotic resistance levels occurs while antibiotic usage is still decreasing.

Decreasing trends in antibiotic usage are assumed to decrease selective pressure and to be followed by decreasing trends in resistance (Allel et al. 2023; Tang et al. 2017), as it was seen in the Dutch livestock for most antibiotics in the past decennium. The relation between AMU and AMR has also been described on the Dutch national level (Dorado‐García et al. 2016; Sanders et al. 2021) and on the European level in animals (Chantziaras et al. 2013) and humans (Allel et al. 2023). However, these studies are mostly ecological and based on a limited level of detail, including for example, the percentage of fully susceptible isolates instead of distinguishing resistance against different antibiotics, or aggregating AMU on a national or sectoral level. Also, several studies have shown that the expected relation between AMU and AMR is complex (Deng et al. 2024) and may vary between for example, countries, sectors and antibiotics. This shows the need for not only ecological studies but also more in‐depth research, including information at the farm level that may give insight into additional factors influencing the relation between AMU and AMR.

In addition however, it seems highly unlikely that complete eradication of AMR for all antibiotics is possible. Due to the mechanisms of AMR spread, AMR might stay present to a certain extent in livestock and the environment even without the presence of selective pressure (Holmes et al. 2016). The lack of evolutionary disadvantage of the resistance genes might result in the persistency of AMR isolates (Ogunlana et al. 2023; Luo et al. 2005). There are several mechanisms that might delay or hinder the reduction in resistance, for example mobile genetic elements such as plasmids harboring resistance genes with addiction systems that make it impossible for the bacteria to survive without the plasmid, resistant bacteria circulating in the environment of the animals that can (re)colonize young animals, or cross‐resistance between antibiotics or co‐selection due to other compounds (Storey et al. 2022; Akwar et al. 2008; Smith et al. 2023). It is expected that Whole Genome Sequencing (WGS)‐based AMR monitoring will help to better understand the complex genomic processes involved in the perseverance and spread of AMR.

The AMR monitoring in food‐producing animals is designed to monitor the level of AMR in indicator bacteria and zoonotic bacteria from a human health perspective. As such, animal samples collected from slaughter are the closest to meat production and are based on EU regulations (European Food Safety Authority (EFSA); European Centre for Disease Prevention and Control (ECDC) 2023). In addition, AMR isolated from meat or other animal products might reflect better the potential consumer risks; however they also depend on factors like cross‐contamination during processing and handling.

If reduction of AMU alone does not result in further reduction of AMR, additional measures might be needed to further reduce AMR. To get more insight in the relation between usage and resistance in the recent years, AMU and AMR should be analysed more in depth, including the possible delayed effect between AMU and resistance development, and the difference in AMR patterns between antibiotics. On‐farm variables as production system, for example, slower grower versus regular broilers, are not included in this study but might be relevant in further research. Long‐term AMR monitoring is necessary to reveal long‐term effects of reduction in AMU in livestock.

## 5 Conclusion

Following a significant decreasing trend in the prevalence of AMR between 2010 and 2018 in broilers, pigs and calves, no significant changes in the prevalence of AMR were observed between 2019 and 2023 for most antibiotics.

Farmers, veterinarians, policymakers, and other stakeholders have made successful efforts in reducing AMU and AMR in the Netherlands. The results of this study however, show the need to improve our understanding of the complex relation between AMU and AMR to identify additional measures to further reduce AMR in livestock.

## Ethics Statement

The authors have nothing to report.

## Conflicts of Interest

The authors declare no conflicts of interest.

## Data Availability

The authors have nothing to report.
